# Paramagnetic Rim Lesions and Choroid Plexus Volume at Diagnosis Are Associated With Cognitive Progression Independent of Relapse and MRI Activity in Early Relapsing–Remitting Multiple Sclerosis

**DOI:** 10.1002/acn3.70448

**Published:** 2026-07-08

**Authors:** Stefano Ziccardi, Damiano Marastoni, Agnese Tamanti, Pietro Biasi, Valentina Visani, Annalisa Colombi, Tom A. Fuchs, Monica Sicchieri, Chiara Eccher, Francesco Guarnaccia, Milena Calderone, Valentina Camera, Francesca Benedetta Pizzini, Roberta Magliozzi, Marco Castellaro, Massimiliano Calabrese

**Affiliations:** ^1^ Neurology Section, Department of Neurosciences, Biomedicine and Movement Sciences University of Verona Verona Italy; ^2^ Multiple Sclerosis Centre University Hospital of Verona Verona Italy; ^3^ Department of Information Engineering University of Padua Padua Italy; ^4^ MS Center Amsterdam, Department of Anatomy and Neurosciences Vrije Universiteit Amsterdam, Amsterdam Neuroscience, Amsterdam UMC, Location VUmc Amsterdam the Netherlands; ^5^ Radiology Unit CMSR Veneto Medica Altavilla Vicentina Vicenza Italy; ^6^ Neuroradiology Unit University of Verona Verona Italy; ^7^ Department of Brain Sciences, Faculty of Medicine Imperial College London London UK

**Keywords:** choroid plexus, cognition, multiple sclerosis, paramagnetic rim lesions, PIRA

## Abstract

Paramagnetic rim lesions (PRLs) and choroid plexus (CP) enlargement reflect smoldering inflammation in multiple sclerosis. Their role in cognitive progression remains unexplored. Eighty‐seven early relapsing–remitting MS patients were enrolled at diagnosis and followed longitudinally. PRLs and CP volume were evaluated at diagnosis using 3 T‐MRI. Cognitive decline over time was classified as cognitive progression independent of relapse/MRI activity (PIRMA). Fifty‐five patients (63.2%) showed meaningful cognitive decline: 44 (80.0%) were cognitive PIRMA. PRLs and CP volume at diagnosis were associated with cognitive PIRMA. PRLs and larger CP reflect cognitive progression independent of clinical/MRI activity, supporting their role as smoldering disease activity markers.

## 1 Introduction

Disability accumulation in multiple sclerosis (MS) mostly occurs independently of relapses (*progression independent of relapse activity*, PIRA), often without concurrent MRI activity [1]. PIRA reflects chronic smoldering inflammation leading to neuroaxonal loss and irreversible CNS damage [1].

While physical disability has traditionally been the primary outcome in MS, increasing evidence indicates that cognitive deficits are frequent and represent a clinically relevant and impactful manifestation of disease worsening. Previous research mainly conceptualized cognitive impairment as a static condition (“preserved” vs. “impaired”); however, greater importance should be placed on the identification and prediction of cognitive decline over time.

Accordingly, the concept of *smoldering‐associated worsening* (SAW) has been proposed, encompassing cognitive worsening, resulting from smoldering pathological processes, which remain unmet therapeutic targets [2]. In particular, a subtle accumulation of disability in terms of cognitive decline has been defined as *cognitive PIRA* [3], occurring often also in the absence of MRI activity (*cognitive progression independent of relapse and MRI activity*, *cognitive PIRMA*) [4]. Cognitive progression independent of relapse/MRI activity is independent from physical disability and is highly predominant also in non‐progressive MS [3, 4, 5]. Silent cognitive progression has been associated with early brain atrophy, while a lower predictive association has been found for clinical measures and for white matter and cortical lesions load [5].

Detecting early surrogate markers of these smoldering processes is an urgent need, as it could enable earlier therapeutic interventions and prevent irreversible disability. Among candidate MRI markers, paramagnetic rim lesions (PRLs) and choroid plexus (CP) enlargement have recently gained increasing attention [6, 7, 8, 9, 10, 11, 12].

PRLs reflect persistent microglial/macrophage activation and iron deposition at the edge of chronic active lesions [1]. They represent a highly specific MS hallmark in approximately half of MS patients and are associated with PIRA, disability accumulation, and brain atrophy [6, 7]. Their inclusion as supportive evidence in the 2024 MS criteria revisions underscores their clinical relevance.

The CP represents a primary site of CSF secretion and an immunologically active blood–CSF barrier [8]. Its enlargement could reflect immune cell trafficking and sustained inflammatory activation and has recently been linked to PIRA [8, 9, 10, 11].

Notably, previous cross‐sectional studies including both relapsing and progressive MS showed that cognitively impaired patients have greater PRL burden and enlarged CP than cognitively preserved, likely reflecting progressive demyelination, neuroaxonal injury, and synaptic degeneration [6, 8, 13]. However, their role in longitudinal cognitive decline remains unclear.

Here, we investigated in early relapsing–remitting MS (RRMS) whether PRLs and CP volume at diagnosis play a role in subsequent cognitive progression independent of any acute disease activity (both relapse and MRI activity).

## 2 Methods

### 2.1 Study Design and Participants

Eighty‐seven RRMS patients were prospectively enrolled at diagnosis in this longitudinal study. At diagnosis, all patients underwent a baseline neurological examination, 3 T‐MRI, and comprehensive neuropsychological assessment. Follow‐up included clinical evaluations every 6 months, annual 3 T‐MRI scans, and cognitive assessments spaced at least annually (in accordance with current recommendations, to reduce practice effects associated with repeated neuropsychological testing). Exclusion criteria were other neurological/psychiatric disorders, substance abuse, or sensory/motor deficits interfering with cognitive assessment.

### 2.2 Neuropsychological Assessment and Monitoring

Neuropsychological evaluations were performed using the Brief Repeatable Battery of Neuropsychological Tests, the Stroop Test, the Trail Making Test, the Phonemic, Semantic, and Alternate Verbal Fluency Tests, the Modified Five‐Point Test, and the Brief Visuospatial Memory Test‐Revised (BVMT‐R) [5].

Meaningful cognitive decline was evaluated using the Reliable Change Index methodology, a conservative method accounting for measurement error or practice effects. We focused on tests with validated RCI values in the Italian population: the Symbol Digit Modalities Test (SDMT), the BVMT‐R, and the Paced Auditory Serial Addition Task (PASAT) [14]. Significant decline was identified in case worsening was higher than RCI threshold in at least one of these tests. Further details are provided in the Supporting Information.

Then, we determined whether cognitive decline occurred independently of relapse activity [3, 4, 5]. To increase specificity, we focused only on patients who were also concomitantly free from MRI activity to be sure to catch cognitive worsening not explained by other acute inflammatory processes [4]. Following prior literature, cognitive decline was classified as cognitive PIRMA if no disease activity (neither clinical nor MRI) occurred within an 18‐month window (between ±9 months before/after the cognitive decline) [3, 4, 5]. If a relapse occurred during this interval, the event was defined as relapse‐associated worsening (RAW).

This 18‐month criterion represents a more conservative approach than the conventional ±3‐month window typically used to confirm relapse‐free status [7]. Given that neurological evaluations and MRI scans are usually performed more frequently than cognitive testing in clinical practice, this extended timeframe ensures greater confidence in confirming relapse‐free intervals. Shorter windows (≤ 6 months) often coincide with a single neurological visit, which may limit accurate detection of relapse activity. Previous studies have also demonstrated that cognitive PIRA/PIRMA occurs more frequently than PIRA based on physical or EDSS progression [3, 5].

### 2.3 MRI Acquisition and Analyses

At baseline (diagnosis), we focused on PRLs and CP volume: PRLs were evaluated on filtered phase images generated from the phase data of the susceptibility‐weighted images (SWI) acquisition, while CP volume was automatically calculated and was normalized for total intracranial volume. Yearly follow‐up MRIs allowed us to evaluate MRI activity (new/gad+ lesions in the time between ±9 months before/after the cognitive decline) [4]. Specific details are provided in the Supporting Information.

### 2.4 Statistical Analyses

Group comparisons (cognitively stable vs. cognitive PIRMA) were performed using Mann–Whitney test analyses with false discovery rate (FDR) correction. Univariable and multivariable logistic regressions were constructed, including PRLs and CP as main predictors and adjusting for potentially associated factors with both cognitive PIRMA and MRI markers (baseline age, sex, EDSS, follow‐up duration, treatment exposures, lesion burden). Significance level was set at *p* < 0.05.

## 3 Results

All patients were longitudinally observed for a minimum of two years (mean ± SD observation period = 6.2 ± 2.4 years) and remained RRMS throughout the study. Baseline clinical and MRI characteristics of the whole sample are summarized in Table 1.

### 3.1 Prevalence of Cognitive Progression Independent of Relapse and MRI Activity

During follow‐up, 55 patients (63.2%) showed meaningful cognitive decline. Among them, the vast majority (44 patients, 80.0%) experienced the decline without temporally associated relapse or MRI activity and were classified as cognitive PIRMA; 8 patients (14.5%) had relapse‐associated worsening, and 3 (5.5%) showed concomitant MRI activity (Figure 1). No significant demographic or clinical differences were observed between cognitively stable patients and those with cognitive PIRMA (Table 1).

**FIGURE 1:**
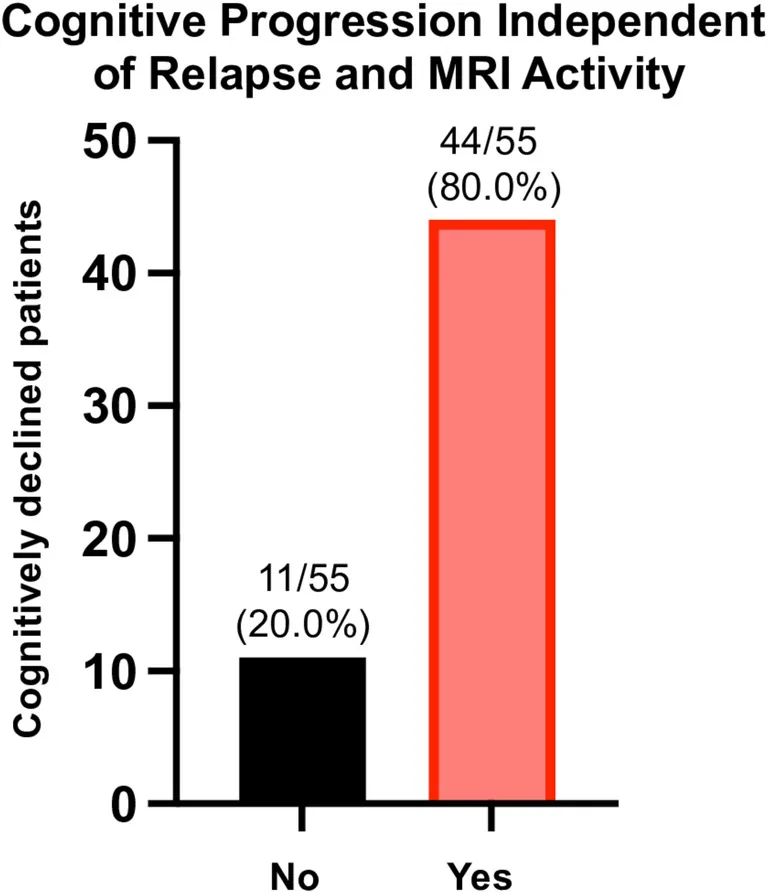
Prevalence of cognitive progression among cognitively declined RRMS patients. Proportion of cognitively‐declined patients who experienced acute disease activity (*n* = 11, in black) or not (*n* = 44, patients with cognitive progression independent of relapse and MRI activity, in red) concomitantly with the cognitive decline.

**TABLE 1:** Clinical, MRI, and neuropsychological characteristics of RRMS patients included in the study, separating cognitively stable patients and patients with cognitive progression independent of relapse and MRI activity.

	Cognitively stable patients	Patients with cognitive progression independent of relapse and MRI activity	*p*‐value
*Demographic and clinical data*			
Age at diagnosis (T0), years	37.2 ± 11.5	40.5 ± 12.2	0.54
Female, *n* (%)	26 (81.3%)	29 (65.9%)	0.56
EDSS at diagnosis (T0)	2.0 [2.0]	2.0 [2.0]	0.50
Baseline DMT, *n* (%)			
Low efficacy	16 (50.0%)	24 (54.5%)	0.13
High efficacy	6 (18.8%)	11 (25.0%)	
None	10 (31.3%)	9 (20.5%)	
DMT switchers over the period of observation, *n* (%)			
Yes	17 (53.1%)	15 (34.1%)	0.19
No	15 (46.9%)	29 (65.9%)	
*3 T MRI data at diagnosis (T0)*			
WML, *n*	8.0 ± 3.1	9.8 ± 4.1	0.06
nCPV, % ICV	0.16 ± 0.03	0.18 ± 0.05	**0.036**
PRLs, n	1.0 [2.0]	1.5 [2.75]	**0.031**
Proportion of patients with ≥ 1PRL	52.0%	85.3%	**0.005**
*Neuropsychological data at diagnosis (T0)*			
Education, years	14.4 ± 2.8	14.3 ± 3.8	0.85
Cognitive status, *n* (%)			
CN	12 (37.5%)	13 (29.5%)	0.08
mCI	14 (43.8%)	12 (27.3%)	
sCI	6 (18.8%)	19 (43.2%)	
SRT‐LTS	47.8 ± 15.2	44.6 ± 15.1	0.62
SRT‐CLTR	41.2 ± 17.0	35.7 ± 18.3	0.55
SRT‐D	9.3 ± 2.6	8.1 ± 2.8	0.30
SPART	23.1 ± 4.2	21.5 ± 5.2	0.55
SPART‐D	8.1 ± 1.8	7.0 ± 2.3	0.28
SDMT	52.9 ± 10.9	50.2 ± 11.7	0.63
PASAT‐3	40.0 ± 11.9	40.3 ± 12.1	0.90
PASAT‐2	31.6 ± 10.0	31.1 ± 10.6	0.86
WLG	26.6 ± 5.5	23.6 ± 6.8	0.25
ST‐EIT	14.7 ± 6.6	15.8 ± 8.4	0.80
ST‐EIE	0.5 ± 1.4	0.6 ± 1.1	0.62
TMT‐A	25.9 ± 11.9	30.7 ± 18.8	0.54
TMT‐B	75.2 ± 24.4	90.4 ± 62.5	0.62
PVF	40.5 ± 10.7	38.8 ± 14.6	0.62
SVF	54.5 ± 10.2	51.6 ± 11.9	0.55
AVF	42.3 ± 9.5	37.1 ± 11.3	0.28
MFPT‐UDs	34.3 ± 10.4	27.8 ± 10.1	0.25
MFPT‐CSs	16.8 ± 14.7	9.9 ± 10.0	0.28
MFPT‐ErrInd	6.9 ± 9.3	9.4 ± 10.8	0.55
BVMT‐R	26.7 ± 6.2	24.8 ± 7.3	0.62
*Neuropsychological data (follow‐up)*			
Follow‐up period of observation, years	5.9 ± 2.5	6.4 ± 2.2	0.52
Numbers of cognitive assessments	3.5 [1.25]	4 [1]	0.47
Years between cognitive assessments	2.6 ± 0.9	2.7 ± 1.0	0.63

*Note:* Continuous data are reported as mean ± SD. Discrete data are reported as median [IQR]. FDR‐corrected *p*‐values are shown.

Abbreviations: AVF = Alternate Verbal Fluency; BVMT‐R = Brief Visuospatial Memory Test‐Revised; CN = Cognitive Normal; DMT = disease modifying treatment; EDSS = Expanded Disability Status Scale; ICV = intracranial volume; mCI = mildly cognitively impaired; MFPT‐CSs = Modified Five Point Test‐Cumulative Strategies; MFPT‐ErrInd = Modified Five Point Test‐Error Index; MFPT‐UDs = Modified Five Point Test‐Unique Designs; nCPV = normalized choroid plexus volume; PASAT‐2 = Paced Auditory Serial Addition Task‐2 s; PASAT‐3 = Paced Auditory Serial Addition Task‐3 s; PRLs = paramagnetic rim lesions; PVF = Phonemic Verbal Fluency; sCI = severely cognitively impaired; SDMT = Symbol Digit Modalities Test; SPART = Spatial Recall Test; SPART‐D = Spatial Recall Test‐Delayed; SRT‐CLTR = Selective Reminding Test‐Consistent Long Term Retrieval; SRT‐D = Selective Reminding Test‐Delayed; SRT‐LTS = Selective Reminding Test‐Long Term Storage; ST‐EIE = Stroop Test‐Effect Interference Error; ST‐EIT = Stroop Test‐Effect Interference Time; SVF = Semantic Verbal Fluency; TMT‐A = Trail Making Test‐A; TMT‐B = Trail Making Test‐B; WLG = Word List Generation; WML = white matter lesions.

### 3.2 Paramagnetic Rim Lesions and Cognitive Progression Independent of Relapse and MRI Activity

Patients who developed cognitive PIRMA during the follow‐up observation period showed at diagnosis a significantly higher number of PRLs compared to cognitively stable patients (1.5 [2.75] vs. 1.0 [2.0]; FDR‐corrected *p* = 0.031) (Figure 2A). No differences were observed in overall WML burden (Table 1).

**FIGURE 2:**
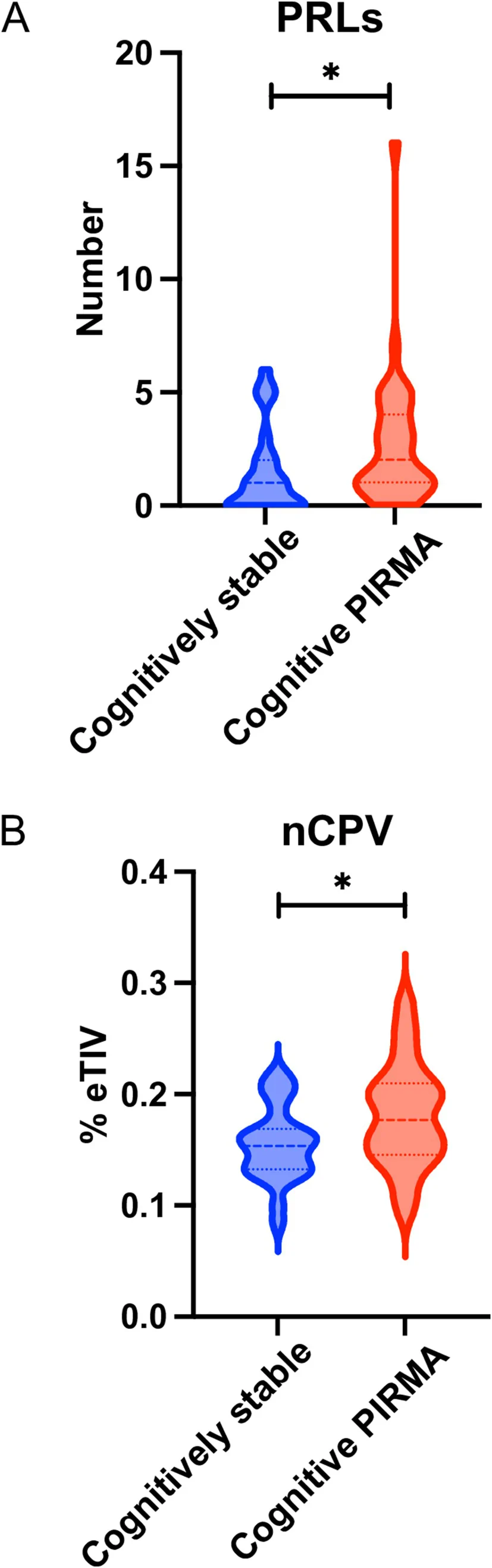
MRI markers of smoldering inflammation and cognitive progression. Comparison of paramagnetic rim lesions (panel A) and choroid plexus volume (panel B) at diagnosis according to the occurrence of cognitive progression independent of relapse and MRI activity over the follow‐up. Cognitively stable individuals are displayed in blue; cognitive PIRMA individuals are displayed in red. PRLs = paramagnetic rim lesions; nCPV = normalized choroid plexus volume; ICV = total intracranial volume; PIRMA = progression independent of relapse and MRI activity.

In the logistic regression models focused on cognitive PIRMA as the clinical outcome, the presence of PRLs (≥ 1) was statistically significant in the univariable model (OR = 5.4, 95% CI = 1.6–18.3, *p* = 0.008) with a modest discriminant ability (AUC = 0.67, 95% CI = 0.55–0.78). We then performed a multivariable model including other key demographic, clinical, and MRI covariates to adjust for their confounding effect (baseline, age, sex, EDSS, treatment exposure, follow‐up duration, and WMLv): PRLs presence resulted as the only statistically significant variable (OR = 5.2, 95% CI = 1.0–26.7, *p* = 0.048) with a good discriminative ability (AUC = 0.80, 95% CI = 0.61–0.92) (Figure 3A and Table S2).

**FIGURE 3:**
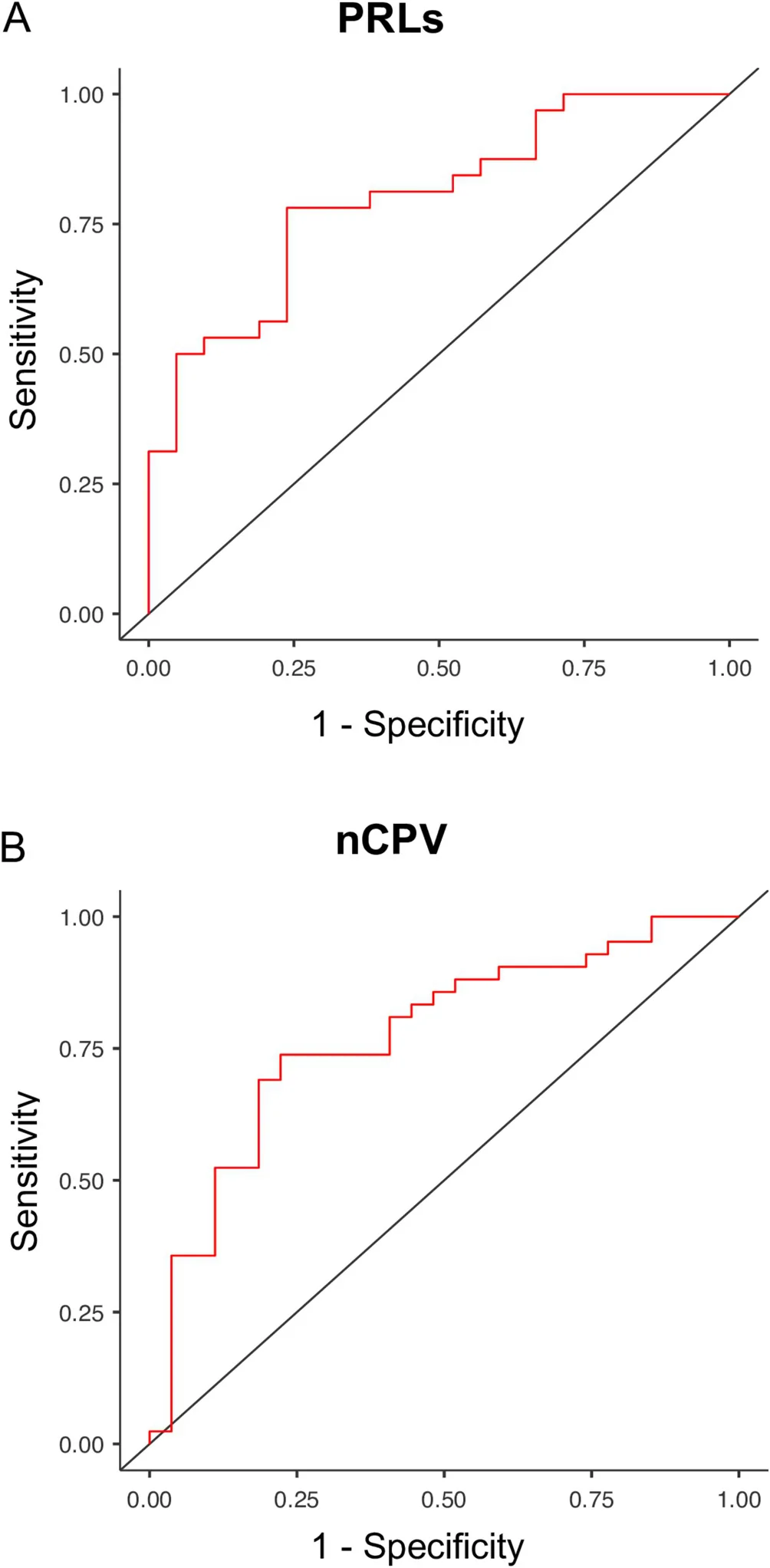
Receiver operating characteristic (ROC) curve for cognitive progression independent of relapse and MRI activity over the follow‐up. The ROC curve illustrated the diagnostic performance of the multivariable models (baseline age, sex, EDSS, follow‐up duration, treatment exposure, lesion burden) with the inclusion of paramagnetic rim lesions (panel A) and choroid plexus volume (panel B) at diagnosis in discriminating between cognitive progression independent of relapse and MRI activity over the follow‐up. The area under the curve (AUC) in panel A was 0.80, and in panel B was 0.77. The diagonal line represents the reference line corresponding to no discriminative ability (AUC = 0.5). PRLs = paramagnetic rim lesions; nCPV = normalized choroid plexus volume.

Conversely, PRLs’ number showed a trend towards significance in the univariable model (*p* = 0.093), which remained not significant after adjusting for key covariates in the multivariable models.

### 3.3 Choroid Plexus and Cognitive Progression Independent of Relapse and MRI Activity

Similarly, normalized CP volume at diagnosis was significantly larger in patients who developed cognitive PIRMA during the follow‐up observation period compared to cognitively stable individuals (0.18% ± 0.05% vs. 0.16% ± 0.03%; FDR‐corrected *p* = 0.036) (Figure 2B).

In the logistic regression models focused on cognitive PIRMA as the clinical outcome, CP volume was statistically significant in the univariable model (OR = 4.8, 95% CI = 1.3–18.3, *p* = 0.021) with a modest discriminant ability (AUC = 0.67, 95% CI = 0.54–0.80). The multivariable model, including other key covariates to adjust for their confounding effect (baseline, age, sex, EDSS, treatment exposure, follow‐up duration), identified CP volume as the only statistically significant variable (OR = 5.1, 95% CI = 1.1–23.1, *p* = 0.034) with a moderate discriminative ability (AUC = 0.77, 95% CI = 0.66–0.89) (Figure 3B and Table S3).

## 4 Discussion

In this longitudinal study on early RRMS, cognitive progression independent of relapse and MRI activity emerged as the most frequent form of cognitive decline [3, 4, 5]. Importantly, we provided preliminary novel evidence that both the presence of PRLs and larger CP at diagnosis could represent useful MRI markers associated with subsequent cognitive PIRMA, supporting their potential role as early imaging markers of smoldering disease activity.

The addition of MRI markers resulted in a meaningful improvement in model discrimination, suggesting incremental prognostic value beyond clinical variables alone. Presence/absence of PRLs, found in a great proportion of patients, similarly to previously published studies [6], reached higher discriminative ability compared to PRLs burden, supporting their applicability in discriminating their presence to have important information on disease status, more effective than the calculation of their exact number, which can have limited feasibility among MS centers.

Both PRLs and CP enlargement reflect chronic compartmentalized inflammation that ultimately led to disability accrual. The persistent microglial activity at the rim of chronic active lesions contributes to neuroaxonal damage and clinical/cognitive disability [6]. Concurrently, a dysfunctional CP at the blood–CSF interface could promote sustained immune cell trafficking and innate immune activation, ultimately leading to a proinflammatory CNS milieu [15].

Cognitive progression could represent a window into smoldering processes that lead to chronic inflammatory processes. Therefore, cognition should be considered and carefully monitored over time to reach a more adequate control on disease evolution and associated processes. Choroid plexus volume and PRLs may represent potential markers of silent cognitive progression.

Study limitations include the modest sample size and the relatively limited follow‐up. The interval between cognitive assessments may have limited the temporal precision in identifying the onset of cognitive decline, as well as the restriction to cognitive tests with available RCI thresholds. Strengths include the homogeneous early RRMS cohort, the regular neuropsychological and MRI follow‐up, and the conservative criterion of cognitive progression adopted, excluding both relapse and focal MRI activity. Future prospective studies will have to evaluate confirmation of cognitive decline over the follow‐up, to include results regarding EDSS‐PIRA, and to perform multiparametric analyses to further clarify the potential complementary and synergistic contribution of MRI proxies when considered together (as suggested by the results of our combined model, where CP volume showed a significant association).

Concluding, cognitive PIRMA is frequent in early MS and is associated with the presence of PRLs and larger CP at diagnosis. These MRI markers merit further investigation as measures to improve early risk stratification and potentially enhance personalized therapeutic strategies aimed at preventing both physical and cognitive subtle disability accumulation.

## Author Contributions

S.Z.: Contributed to drafting/revision of the manuscript for content, including medical writing for content; study concept or design; major role in the acquisition of data; and analysis or interpretation of data. D.M.: Contributed to drafting/revision of the manuscript for content, including medical writing for content; study concept or design; major role in the acquisition of data; and analysis or interpretation of data. A.T.: Contributed to drafting/revision of the manuscript for content, including medical writing for content, and analysis or interpretation of data. P.B.: Contributed to drafting/revision of the manuscript for content, including medical writing for content; major role in the acquisition of data; and analysis or interpretation of data. V.V.: Contributed to drafting/revision of the manuscript for content, including medical writing for content; major role in the acquisition of data; and analysis or interpretation of data. A.C.: Contributed to drafting/revision of the manuscript for content, including medical writing for content; major role in the acquisition of data; and analysis or interpretation of data. T.A.F.: Contributed to drafting/revision of the manuscript for content, including medical writing for content, and analysis or interpretation of data. M.S.: Contributed to drafting/revision of the manuscript for content, including medical writing for content, and analysis or interpretation of data. C.E.: Contributed to drafting/revision of the manuscript for content, including medical writing for content, and analysis or interpretation of data. F.G.: Contributed to drafting/revision of the manuscript for content, including medical writing for content, and analysis or interpretation of data. M.C.: Contributed to drafting/revision of the manuscript for content, including medical writing for content, and analysis or interpretation of data. V.C.: Contributed to drafting/revision of the manuscript for content, including medical writing for content, and analysis or interpretation of data. F.B.P.: Contributed to drafting/revision of the manuscript for content, including medical writing for content, and analysis or interpretation of data. R.M.: Contributed to drafting/revision of the manuscript for content, including medical writing for content, and analysis or interpretation of data. M.C.: Contributed to drafting/revision of the manuscript for content, including medical writing for content; major role in the acquisition of data; and analysis or interpretation of data. M.C.: Contributed to drafting/revision of the manuscript for content, including medical writing for content; study concept or design; major role in the acquisition of data; and analysis or interpretation of data.

## Funding

The authors have nothing to report.

## Ethics Statement

The local Ethics committee approved the study (A.O.U.I. Verona, protocol no. 66418).

## Consent

Informed consent was collected from all participants.

## Conflicts of Interest

S.Z.: Received research support from Novartis. D.M.: Received research support and/or honoraria for speaking and funds for travel from Roche, Sanofi‐Genzyme, Merck‐Serono, Biogen Idec, and Novartis, and receives research support from #NEXTGENERATIONEU (NGEU) of the Italian Ministry of University and Research (MUR), National Recovery and Resilience Plan (NRRP), project MNESYS (PE0000006). A.T.: Received research support from Merck. P.B.: Nothing to disclose. V.V.: Nothing to disclose. A.C.: Nothing to disclose. T.A.F.: Received research support from the European Committee for Treatment and Research in Multiple Sclerosis and consulting fees for Click Therapeutics. M.S.: Nothing to disclose. C.E.: Nothing to disclose. F.G.: Nothing to disclose. M.C.: Nothing to disclose. V.C.: Received research grant from the European Charcot Foundation, received support for scientific meetings from Biogen, Janssen, Novartis, BMS, Roche, and received advisory board and speaking honoraria from Novartis. F.B.P.: Nothing to disclose. R.M.: Partially funded by Italian M.S. Society (grant FISM 2023/R‐Single/038). M.C.: Nothing to disclose. M.C.: Received support from the Italian Ministry of Health and received consulting and/or lecture fees and/or travel grants from Roche, Biogen Idec, Sanofi Genzyme, Novartis, and Merck Serono.

## Supporting information


**Supporting Information:** Methods.

**Table S1:** Clinical, MRI, and cognitive characteristics of RRMS patients included in the study.

**Table S2:** Multivariable logistic regression model including the PRLs presence.

**Table S3:** Multivariable logistic regression model including the normalized CP volume.

## Data Availability

The data that support the findings of this study are available from the corresponding author upon reasonable request.
