# Supplementary material for: Paramagnetic Rim Lesions and Choroid Plexus Volume at Diagnosis Are Associated With Cognitive Progression Independent of Relapse and MRI Activity in Early Relapsing–Remitting Multiple Sclerosis
Source: Ann Clin Transl Neurol. 2026 Jul 8:10.1002/acn3.70448. Online ahead of print. doi: 10.1002/acn3.70448 (PMC13394531; doi:10.1002/acn3.70448)
Supplement: Supplementary file 1 — Supporting Information: Methods. [file ACN3-9999-0-s004.docx]

**Supplementary Materials – Methods**

*Neuropsychological assessment and monitoring*

All assessments were carried out during clinically stable periods, defined as no relapses or corticosteroid treatment within the preceding 30 days, and were performed in conjunction with a neurological examination and MRI scans.^1^

Raw scores were adjusted for age, education, and gender based on Italian normative data for each test. Corrected scores below the clinical cut-off (fifth percentile) were classified as failed. Cognitive status of each patient was determined considering all neuropsychological tests administered using previously published conservative criterion^1^: “Cognitively Normal” (CN) in case no tests were found below the clinical cut-off, and “Cognitively Impaired (CI) in case at least one test was found below the clinical cut-off (then further subdivided into “mildly impaired” (mCI, up to 2 failed subtests), or “severely impaired” (sCI, 3 or more failed subtests).

Cognitive decline was determined using the **Reliable Change Index (RCI)**, a conservative, standardized method designed to detect genuine cognitive changes exceeding expected variability due to measurement error or practice effects.^2^ Compared to conventional group-level analyses, this method applies a strict threshold for identifying reliable change.^2,3^ Cognitive decline was operationalized through a **sliding-window approach**, whereby performance at each evaluation was re-baselined relative to the immediately preceding assessment.^4-6^

*MRI acquisition*

At diagnosis, all participants underwent 3T MRI scanning at the Neuroradiology Unit, University Hospital of Verona, performed with a Phillips Achieva scanner. Quantitative analyses included normalized choroid plexus (CP) volume, paramagnetic rim lesion (PRL) count, white matter lesion number and volume. All images underwent quality control to exclude artifacts and scanner inhomogeneities.

The MRI protocol included the following sequences:

- 3D T1-weighted Turbo Field Echo (TFE) invert prepulse (repetition time, TR = 8.4 ms; echo time, TE = 3.7 ms; inversion time, TI = 1050 ms; flip angle = 8°; voxel size = 1×1×1 mm³; field of view, FOV = 240×240×180 mm^3^; matrix size = 240×240×180; number of excitations, NEX = 1; acquisition time, TA = 05:05);
- 3D T2-weighted Turbo Spin Echo (TSE) Fluid Attenuated Inversion Recovery, FLAIR (TR = 8000 ms; TE = 288 ms; TI = 2356 ms; flip angle = 90°; voxel size = 1×1×1 mm³; FOV = 240×240×180 mm^3^; matrix size = 240×240×180; NEX = 1; TA = 04:48);
- 3D TSE Double Inversion Recovery, DIR (TR = 5500 ms; TE = 275 ms; TI = 2550 ms; delay = 450 ms; flip angle = 90°; voxel size = 1×1×1 mm³; FOV = 240×240×180 mm^3^; matrix size = 240×240×180; NEX = 3; TA = 10:49);
- 3D Echo Planar Imaging, Susceptibility-Weighted Imaging, EPI SWI (TR = 54 ms; TE = 29 ms; flip angle = 10°; EPI factor = 11; voxel = 0.55×0.55×0.55 mm³, FOV = 220×220×184.8 mm^3^; matrix size = 400×400×336; NEX = 2; TA = 05:27).

*MRI processing*

White matter lesions were segmented automatically using Lesion Segmentation Tool (LST)^8^ on FLAIR images, thus obtaining a T2-hyperintense white matter lesion number (WMLn) (defined by the connected components of the lesion mask).

CP within the lateral ventricles was automatically segmented using **ASCHOPLEX** (https://gitlab.dei.unipd.it/fair/aschoplex),^9^ a deep learning based toolbox trained on non-contrast-enhanced 3D T1-weighted images^10^ acquired with the same scanner used in this study. ASCHOPLEX was previously validated in a cohort of RRMS patients included in this investigation and in a restricted group of healthy controls. In the ASCHOPLEX validation study that was carried out on a dataset including that used in this study, manual segmentations were used to train and evaluate the accuracy of the automatic segmentation. The T1-weighted sequence was lesion-filled (using the previously obtained lesion mask) and processed with **FreeSurfer v7.3.1^11^** to estimate total Intracranial Volume (eTIV), used to normalize CP volume (CPv) and control for inter-individual brain size variability.^9,10^

For the detection and quantification of PRL lesions, filtered phase images were generated from the phase data of the susceptibility-weighted images (SWI) acquisition using Laplacian phase unwrapping followed by high-pass Gaussian filtering.^12^ T1-weighted and FLAIR images were coregistered to the SWI space using ANTs.^13^ ITK-SNAP workspaces were created to enable simultaneous visualization of the filtered phase images alongside the coregistered T1-weighted and FLAIR images. Supratentorial MS lesions were assessed by visual inspection and classified as paramagnetic rim–positive when a hypointense rim was observed at the lesion edge on phase-filtered images, in accordance with recently proposed NAIMS PRL criteria.^14^ PRLs were independently evaluated by two expert raters, who were blinded to clinical outcomes.

Evaluation of new/gad+ lesions over follow-up in the period of observation, associated with neuropsychological assessments, was retrospectively derived from radiological reports.

**Supplementary Materials – Results**

*Paramagnetic Rim Lesions Identification*

In the PRLs identification, an agreement of 94% was reached between the two expert raters blinded to clinical outcomes, with a Cohen’s k of 0.86, indicating excellent agreement beyond chance.

*Paramagnetic Rim Lesions, Choroid Plexus, and Cognitive Progression Independent of Relapse and MRI Activity*

We conducted multivariate models including together both PRLs and CP volume data, in addition to other clinical, demographic, and MRI variables used in the main models (age, sex, EDSS, DMT at baseline, DMT switch, follow-up duration, WML volume), to evaluate their synergic association with cognitive progression independent of both relapse and MRI activity. Results showed that choroid plexus volume emerged with statistical significance, both in the model considering PRLs number (CP: OR=10.1, 95% CI=1.5-70.3, OR= p=0.019) and in the one considering PRLs presence (CP: OR=10.2, 95% CI =1.4-75.4, p=0.023), with good discriminative ability (AUC=0.81, 95%CI=0.70-0.93, and AUC=0.84, 95%CI=0.73-0.95, respectively). PRLs presence showed a trend toward statistical significance (OR=4.6, 95% CI=0.9-25.1, p=0.075).

**Supplementary Materials - References**

1. Ziccardi S, Crescenzo F, Guandalini M, et al. Early regional cerebral grey matter damage predicts long-term cognitive impairment phenotypes in multiple sclerosis: a 20-year study. *Brain Commun*. 2024;6(6):fcae355. doi:10.1093/braincomms/fcae355
2. Weinstock Z, Morrow S, Conway D, et al. Interpreting change on the Symbol Digit Modalities Test in people with relapsing multiple sclerosis using the reliable change methodology. *Mult Scler*. 2022;28(7):1101-1111. doi:10.1177/13524585211049397
3. Strober LB, Bruce JM, Arnett PA, et al. A much needed metric: Defining reliable and statistically meaningful change of the oral version Symbol Digit Modalities Test (SDMT). *Mult Scler Relat Disord*. 2022;57:103405. doi:10.1016/j.msard.2021.103405
4. Fuchs TA, Schoonheim MM, Zivadinov R, et al. Cognitive progression independent of relapse in multiple sclerosis. *Mult Scler*. 2024;30(11-12):1468-1478. doi:10.1177/13524585241256540
5. Ziccardi S, Fuchs TA, Martinelli L, et al. Cognition beyond relapses: cognitive progression independent of relapse activity reflects early smoldering neurodegeneration in multiple sclerosis. *Mult Scler Relat Disord*. Published online February 16, 2026. doi:10.1016/j.msard.2026.107080
6. Ziccardi S, Fuchs TA, Guandalini M, Marastoni D, Benedict RHB, Calabrese M. Cognitive Progression Independent of Relapse and MRI Activity. *Neurology Open Access.* 2025;1;1. doi: 10.1212/WN9.0000000000000005
7. Müller J, Cagol A, Lorscheider J, et al. Harmonizing Definitions for Progression Independent of Relapse Activity in Multiple Sclerosis: A Systematic Review. *JAMA Neurol*. 2023;80(11):1232-1245. doi:10.1001/jamaneurol.2023.3331
8. Schmidt, P., Gaser, C., Arsic, et al. An automated tool for detection of FLAIR-hyperintense white-matter lesions in multiple sclerosis. *Neuroimage*. 2012;59(4),3774-3783. doi:10.1016/j.neuroimage.2011.11.032
9. Visani V, Veronese M, Pizzini FB, et al. ASCHOPLEX: A generalizable approach for the automatic segmentation of choroid plexus. *Comput Biol Med*. 2024;182:109164. doi:10.1016/j.compbiomed.2024.109164
10. Visani V, Pizzini FB, Natale V, et al. Choroid plexus volume in multiple sclerosis can be estimated on structural MRI avoiding contrast injection. *Eur Radiol Exp*. 2024;8(1):33. doi:10.1186/s41747-024-00421-9
11. Fischl B, Dale AM. Measuring the thickness of the human cerebral cortex from magnetic resonance images. *Proc Natl Acad Sci U S A*. 2000;97(20):11050-11055. doi:10.1073/pnas.200033797
12. Absinta M, Sati P, Fechner A, Schindler MK, Nair G, Reich DS. Identification of Chronic Active Multiple Sclerosis Lesions on 3T MRI. *AJNR Am J Neuroradiol*. 2018;39(7):1233-1238. doi:10.3174/ajnr.A5660
13. Avants BB, Yushkevich P, Pluta J, et al. The optimal template effect in hippocampus studies of diseased populations. *Neuroimage*. 2010;49(3):2457-2466. doi:10.1016/j.neuroimage.2009.09.062
14. Bagnato F, Sati P, Hemond CC, et al. Imaging chronic active lesions in multiple sclerosis: a consensus statement. *Brain*. 2024;147(9):2913-2933. doi:10.1093/brain/awae013
