# Supplementary material for: Paramagnetic Rim Lesions and Choroid Plexus Volume at Diagnosis Are Associated With Cognitive Progression Independent of Relapse and MRI Activity in Early Relapsing–Remitting Multiple Sclerosis
Source: Ann Clin Transl Neurol. 2026 Jul 8:10.1002/acn3.70448. Online ahead of print. doi: 10.1002/acn3.70448 (PMC13394531; doi:10.1002/acn3.70448)
Supplement: Supplementary file 2 — Table S1: Clinical, MRI, and cognitive characteristics of RRMS patients included in the study. [file ACN3-9999-0-s002.docx]

|  | **RRMS patients (n=87)** |
| --- | --- |
| *Demographic and clinical data* | |
| Age at diagnosis (T0), years | 39.0 ± 11.8 |
| Female, n (%) | 65 (74.7%) |
| EDSS at diagnosis (T0) | 2.0 [2.0] |
| Baseline DMT, n (%)  Low efficacy  High efficacy  None | 46 (52.9%)  20 (23.0%)  21 (24.1%) |
| DMT switchers over the period of observation, n (%)  Yes  No | 39 (44.8%)  48 (55.2%) |
| *3T MRI data at diagnosis (T0)* | |
| WML, n | 9.3 ± 4.2 |
| nCPV, % ICV | 0.17 ± 0.04 |
| PRLs, n | 1.0 [3.0] |
| Proportion of patients with ≥1PRL | 70.1% |
| *Neuropsychological data at diagnosis (T0)* | |
| Education, years | 14.3 ± 3.6 |
| Cognitive status, n (%)  CN  mCI  sCI | 31 (35.6%)  27 (31.0%)  29 (33.3%) |
| SRT-LTS | 45.8 ± 15.0 |
| SRT-CLTR | 38.2 ± 17.9 |
| SRT-D | 8.6 ± 2.8 |
| SPART | 22.1 ± 4.6 |
| SPART-D | 7.4 ± 2.1 |
| SDMT | 51.1 ± 12.0 |
| PASAT-3 | 39.7 ± 12.3 |
| PASAT-2 | 31.0 ± 10.1 |
| WLG | 25.4 ± 6.7 |
| ST-EIT | 15.8 ± 7.8 |
| ST-EIE | 0.6 ± 1.3 |
| TMT-A | 29.2 ± 15.5 |
| TMT-B | 84.6 ± 48.9 |
| PVF | 40.5 ± 13.8 |
| SVF | 52.9 ± 11.5 |
| AVF | 39.7 ± 11.6 |
| MFPT-UDs | 30.3 ± 10.2 |
| MFPT-CSs | 12.8 ± 12.1 |
| MFPT-ErrInd | 8.7 ± 10.0 |
| BVMT-R | 24.8 ± 7.3 |
| *Neuropsychological data (follow-up)* | |
| Follow-up period of observation, years | 6.2 ± 2.4 |
| Numbers of cognitive assessments | 4 [2] |
| Years between cognitive assessments | 2.7 ± 0.9 |

**Table S1. Clinical, MRI and cognitive characteristics of RRMS patients included in the study.**

Continuous data are reported as mean ± SD. Discrete data are reported as median [IQR].

*EDSS = Expanded Disability Status Scale; DMT = disease modifying treatment; WML = white matter lesions*; *nCPV = normalized choroid plexus volume; ICV = intracrainial volume; PRLs = paramagnetic rim lesions; CN = Cognitive Normal; mCI = mildly cognitively impaired; sCI = severely cognitively impaired; SRT-LTS = Selective Reminding Test-Long Term Storage; SRT-CLTR = Selective Reminding Test-Consistent Long Term Retrieval; SRT-D = Selective Reminding Test-Delayed; SPART = Spatial Recall Test; SPART-D = Spatial Recall Test-Delayed; SDMT = Symbol Digit Modalities Test; PASAT-3 = Paced Auditory Serial Addition Task-3 seconds; PASAT-2 = Paced Auditory Serial Addition Task-2 seconds; WLG = Word List Generation; ST-EIT = Stroop Test-Effect Interference Time; ST-EIE = Stroop Test-Effect Interference Error; TMT-A = Trail Making Test-A; TMT-B = Trail Making Test-B; PVF = Phonemic Verbal Fluency; SVF = Semantic Verbal Fluency; AVF = Alternate Verbal Fluency; MFPT-UDs = Modified Five Point Test-Unique Designs; MFPT-CSs = Modified Five Point Test-Cumulative Strategies; MFPT-ErrInd = Modified Five Point Test-Error Index; BVMT-R = Brief Visuospatial Memory Test-Revised.*
