# Supplementary material for: Paramagnetic Rim Lesions and Choroid Plexus Volume at Diagnosis Are Associated With Cognitive Progression Independent of Relapse and MRI Activity in Early Relapsing–Remitting Multiple Sclerosis
Source: Ann Clin Transl Neurol. 2026 Jul 8:10.1002/acn3.70448. Online ahead of print. doi: 10.1002/acn3.70448 (PMC13394531; doi:10.1002/acn3.70448)
Supplement: Supplementary file 3 — Table S2: Multivariable logistic regression model including the PRLs presence. [file ACN3-9999-0-s003.docx]

| **Variable** | **ORs (95% CIs)** | **p-value** |
| --- | --- | --- |
| **PRLs at diagnosis (T0)** | **5.2 (1.0-26.7)** | **0.048** |
| WMLv | 1.0 (1.0-1.0) | 0.28 |
| Age at diagnosis (T0) | 1.1 (1.0-1.1) | 0.07 |
| Sex | 0.2 (0.05-1.3) | 0.10 |
| EDSS at diagnosis (T0) | 1.0 (0.6-2.0) | 0.90 |
| DMT at diagnosis (T0) | 1.3 (0.2-8.1) | 0.76 |
| DMT switch over follow-up | 0.4 (0.07-2.1) | 0.27 |
| Follow-up duration | 1.2 (0.8-1.7) | 0.44 |

**Table S2. Multivariable logistic regression model including the PRLs presence.**

The outcome variable was the accumulation of cognitive progression independent of relapse and MRI activity vs cognitively stable patients. Models were adjusted for baseline age, sex, EDSS, WML volume, treatment exposure, and follow-up duration. Reference categories for categorical variables were: no PRLs, male sex, low efficacy DMT at diagnosis, no DMT switch over follow-up. Odds ratios (ORs) with 95% confidence intervals (CIs) are reported.

*PRLs = paramagnetic rim lesions; WMLv = white matter lesion volume; EDSS = Expanded Disability Status Scale; DMT = disease modifying treatment; ORs = odds ratios; CIs = confidence intervals*
