# Supplementary material for: Paramagnetic Rim Lesions and Choroid Plexus Volume at Diagnosis Are Associated With Cognitive Progression Independent of Relapse and MRI Activity in Early Relapsing–Remitting Multiple Sclerosis
Source: Ann Clin Transl Neurol. 2026 Jul 8:10.1002/acn3.70448. Online ahead of print. doi: 10.1002/acn3.70448 (PMC13394531; doi:10.1002/acn3.70448)
Supplement: Supplementary file 4 — Table S3: Multivariable logistic regression model including the normalized CP volume. [file ACN3-9999-0-s001.docx]

| **Variable** | **ORs (95% CIs)** | **p-value** |
| --- | --- | --- |
| **nCPv at diagnosis (T0)** | **5.1 (1.1-23.1)** | **0.034** |
| Age at diagnosis (T0) | 1.0 (1.0-1.1) | 0.41 |
| Sex | 0.3 (0.1-1.1) | 0.07 |
| EDSS at diagnosis (T0) | 1.1 (0.6-1.9) | 0.77 |
| DMT at diagnosis (T0) | 1.1 (0.2-5.4) | 0.89 |
| DMT switch over follow-up | 0.4 (0.1-1.6) | 0.22 |
| Follow-up duration | 1.1 (0.9-1.5) | 0.32 |

**Table S3. Multivariable logistic regression model including the normalized CP volume.**

The outcome variable was the accumulation of cognitive progression independent of relapse and MRI activity vs cognitively stable patients. Models were adjusted for baseline age, sex, EDSS, treatment exposure, and follow-up duration. Reference categories for categorical variables were: male sex, low efficacy DMT at diagnosis, no DMT switch over follow-up. Odds ratios (ORs) with 95% confidence intervals (CIs) are reported.

*nCPv = normalized choroid plexus volume; EDSS = Expanded Disability Status Scale; DMT = disease modifying treatment; ORs = odds ratios; CIs = confidence intervals*
